# Supplementary material for: Cost-utility analysis of adding abiraterone acetate plus prednisone/prednisolone to long-term hormone therapy in newly diagnosed advanced prostate cancer in England: Lifetime decision model based on STAMPEDE trial data
Source: PLoS One. 2022 Jun 2;17(6):e0269192. doi: 10.1371/journal.pone.0269192 (PMC9162346; doi:10.1371/journal.pone.0269192)
Supplement: S1 File — (PDF) [file pone.0269192.s002.pdf]

Cost-utility analysis of adding abiraterone acetate plus prednisone/prednisolone to long-term hormone therapy in newly diagnosed advanced prostate cancer: lifetime decision model in England based on STAMPEDE trial data

Caroline S Clarke*, Rachael M Hunter, Andrea Gabrio, Christopher D Brawley, Fiona C Ingleby, David P Dearnaley, David Matheson, Gerhardt Attard, Hannah L Rush, Rob J Jones, William Cross, Chris Parker, J Martin Russell, Robin Millman, Silke Gillessen, Zafar Malik, Jason F Lester, James Wylie, Noel W Clarke, Mahesh KB Parmar, Matthew R Sydes, Nicholas D James on behalf of the STAMPEDE investigators

* Corresponding author: caroline.clarke@ucl.ac.uk (CSC), Research Department of Primary Care and Population Health, University College London, London, UK.

Supporting Information

File 1

Relating to *Methods: Trial-based analysis methods: survival analysis* section in main manuscript

Parametric survival curves were modelled jointly or separately for the 25 transitions using trial data [1] [2] [3] [4], to allow estimation of lifetime event rates in the lifetime model. The baseline variables assessed for model inclusion are listed in Table S1.

Table S1. Number and description of categories for baseline patient and disease characteristics assessed for inclusion (although not necessarily included) in trial-based survival models.

Variable	Number of categories	Category descriptions
**Baseline disease status**		
Tumour stage (T)	4	≤T2 | T3 | T4 | TX
Lymph node status (N)	3	N0 | N+ | NX
Metastasis (M)	2	M0, M1 lymph | M1 bone, visceral
Visceral metastasis	2	Y | N
Lymph node metastasis	2	Y | N
Gleason score	3	≤7 | ≥8 | unknown (higher is worse)
WHO performance status	2	0 | 1 or 2 (higher is worse)
Prostate specific antigen level	5	Quintiles (higher is worse)
Radiotherapy plans	2	Planned | not planned
**Demographics**		
Age group (years)	4	<60 | 60-64 | 65-69 | ≥70
**Treatment Pathway changes**		
Geography	2	England | not England
Arm H open	2	Before arm H | during arm H
**Transitions in model of time to first event**		
From HS1-3 to HS8, i.e. first event is death from prostate cancer	2	Y | N
From HS1/1/2 to HS5/7/7 respectively, i.e. first event is disease progression with worsening metastases	2	Y | N

References

[1] N. Latimer, "Survival Analysis for Economic Evaluations Alongside Clinical Trials—Extrapolation with Patient-Level Data: Inconsistencies, Limitations, and a Practical Guide," *Medical Decision Making,* vol. 33, no. 6, pp. 743-754, 2013.

[2] C. Williams, J. Lewsey, A. Briggs and D. Mackay, "Cost-Effectiveness Analysis in R Using a Multi-state Modeling Survival Analysis Framework: A Tutorial," *Medical Decision Making,* vol. 37, no. 4, pp. 340-352, 2016.

[3] H. Putter, "Special Issue about Competing Risks and Multi-State Models," *Journal of Statistical Software,* vol. 38, no. 1, 2011.

[4] H. Putter, J. van der Hage, G. de Bock, R. Elgalta and C. van de Velde, "Estimation and Prediction in a Multi-State Model for Breast Cancer," *Biometrical Journal,* vol. 48, no. 3, pp. 366-380, 2006.
