# Supplementary material for: Cost-utility analysis of adding abiraterone acetate plus prednisone/prednisolone to long-term hormone therapy in newly diagnosed advanced prostate cancer in England: Lifetime decision model based on STAMPEDE trial data
Source: PLoS One. 2022 Jun 2;17(6):e0269192. doi: 10.1371/journal.pone.0269192 (PMC9162346; doi:10.1371/journal.pone.0269192)
Supplement: S2 File — (PDF) [file pone.0269192.s003.pdf]

Cost-utility analysis of adding abiraterone acetate plus prednisone/prednisolone to long-term hormone therapy in newly diagnosed advanced prostate cancer: lifetime decision model in England based on STAMPEDE trial data

Caroline S Clarke*, Rachael M Hunter, Andrea Gabrio, Christopher D Brawley, Fiona C Ingleby, David P Dearnaley, David Matheson, Gerhardt Attard, Hannah L Rush, Rob J Jones, William Cross, Chris Parker, J Martin Russell, Robin Millman, Silke Gillessen, Zafar Malik, Jason F Lester, James Wylie, Noel W Clarke, Mahesh KB Parmar, Matthew R Sydes, Nicholas D James on behalf of the STAMPEDE investigators

* Corresponding author: caroline.clarke@ucl.ac.uk (CSC), Research Department of Primary Care and Population Health, University College London, London, UK.

Supporting Information

File 2

Relating to *Methods: Trial-based analysis: Costs* section in main manuscript

Costs captured in the trial and used in the lifetime model were calculated from amounts and frequencies of therapies and procedures reported during the trial, with unit costs applied from the BNF and published NHS reference costs, for medications and any delivery or preparation costs for chemotherapy or radiotherapy. Dose information was missing for most medications.

Unit costs for unscheduled visits are given in Table S2. For primary care costs, it was assumed that the visit was in person to a General Practitioner (GP). Outpatient costs used the mean unit cost for urology outpatient visits. For inpatient costs, the non-elective short-stay healthcare resource group (HRG) cost was applied if length of stay was 0-1 days; the non-elective long-stay cost was applied for stays of 2 days or longer; and for stays longer than the HRG average trim point, additional days were costed as excess bed days. Means of costs across the currency codes LB06N, LB06P, LB06Q, LB06R, and LB06S (Kidney, Urinary Tract or Prostate Neoplasms, without Interventions) were used.

Applied unit costs are given for chemotherapy in in Table S3, and for procedures in Table S4. NHS Reference Cost codes used for chemotherapy delivery costs were SB12Z, SB14Z and SB15Z.

Preparation costs for radiotherapy were according to HRG group SC41Z for Intensity Modulated Radiation Therapy (IMRT) only, SC52Z for Conformal RT only, and delivery costs were the mean of SC22Z, SC23Z, and SC31Z, for both IMRT and Conformal.

Table S2. Unit costs for unscheduled primary and secondary care visits.

Category	Unit cost	Source
GP visits	£37.40	PSSRU 2018
Outpatient attendances	£109.83	NHS Reference Costs 2017-18
Non-elective short stay	£536.85	NHS Reference Costs 2017-18
Non-elective long stay	£3,328.20	NHS Reference Costs 2017-18
Excess bed day	£389.99	NHS Reference Costs 2017-18
Weighted mean trim point	33	NHS Reference Costs 2017-18 (Annex A)

Table S3. Unit costs for chemotherapeutic agents, including delivery and stoppage costs, NHS Reference Costs 2017-18.

Chemotherapeutic agent	Dose	Daily cost (£)	Delivery cost per cycle (£)	Cycle length (days)	Stoppage cost (£)
Cabazitaxel	25 mg/m^2/21d, IV	155.91	198.19	21	104.79
Carboplatin	400 mg/m^2/28d	29.43	247.74	28	130.98
Etoposide	3x120 mg/m^2/21d, IV	57.01	999.20	21	130.98
Cisplatin	100 mg/m^2/28d, IV	3.69	247.74	28	130.98
Docetaxel	75 mg/m^2/21d	28.64	247.74	21	207.39
Mitoxantrone	14 mg/m^2 /21d	2.45	374.52	21	87.32

Table S4. Unit costs for procedures, including orchidectomy, NHS Reference Costs 2017-18.

Name of procedure	Total unit cost of procedure (£, 2017-18 NHS Reference Costs and clinical advice)	NHS Reference Cost code	Source
Orchidectomy	457.49	n/a	Lord et al. [1], adjusted to 2017/18 prices
Urinary catheter	1,262.46	LB18Z	NHS Ref Costs 2017/18
Nephrostomy	1,349.59	YL10Z, YL11Z, YL12Z	NHS Ref Costs 2017/18
TURP	3,016.86	LB25D, LB25E, LB25F	NHS Ref Costs 2017/18
Intramedullary nail	3,439.98	HD39D, HD39E, HD39F, HD39G, HD39H	NHS Ref Costs 2017/18
Salvage prostatectomy	6,415.21	LB22Z	NHS Ref Costs 2017/18
Lobectomy	6,452.53	DZ02H, DZ02J, DZ02K, DZ63A, DZ63B, DZ63C, DZ64A, DZ64B, DZ64C	NHS Ref Costs 2017/18
Bowel obstruction	6,653.48	FF32A, FF32B, FF32C, FF33A, FF33B	NHS Ref Costs 2017/18
Spinal cord surgery	10,599.89	HC28H	NHS Ref Costs 2017/18

Severe Adverse Events

Cardiovascular and musculoskeletal SAEs are the most common SAEs for AAP [2]. Trial SAEs were captured including a brief free-text description of the event, but not always specifying exact treatment, thus limiting information available to calculate costs. They were also partially observed via the unscheduled visits data, leading to possible double counting. Therefore, a premium was calculated for cardiac SAEs (myocardial infarction, stroke, transient ischaemic attack, heart failure, angina) and musculoskeletal SAEs (spinal cord compression, long bone fracture, pathological fracture), as the difference between the mean NHS Reference unit cost for treating each type of SAE, and the mean trial-based cost for an unscheduled visit recorded on the same day as a reported SAE, applied as a flat rate across all participants according to SAE rate per arm. Further detailed information on unit costs are given in Supporting Information, section 2.

The mean SAE premium calculated was a flat cost per patient of £137.78 and £72.41 for cardiac SAEs in the AAP+SOC and SOC-only arms respectively, and £258.03 and £153.66 for musculoskeletal SAEs in the AAP+SOC and SOC-only arms respectively.

References

[1] J. Lord, S. Willis, J. Eatock, P. Tappenden, M. Trapero-Bertran, A. Miners, C. Crossan, M. Westby, A. Anagnostou, S. Taylor, I. Mavranezouli, D. Wonderling, P. Alderson and F. Ruiz, "Economic modelling of diagnostic and treatment pathways in National Institute for Health and Care Excellence clinical guidelines: the Modelling Algorithm Pathways in Guidelines (MAPGuide) project," *Health Technology Assessment,* vol. 17, no. 58, 2013.

[2] R. B. Moreira, M. Debiasi, E. Francini, P. V. Nuzzo, G. de Velasco, F. C. Maluf, A. P. Fay, J. Bellmunt, T. K. Choueiri and F. A. Schutz, "Differential side effects profile in patients with mCRPC treated with abiraterone or enzalutamide: a meta-analysis of randomized controlled trials," *Oncotarget,* vol. 8, no. 48, pp. 84572-84578, 2017.
