# Supplementary material for: Cost-utility analysis of adding abiraterone acetate plus prednisone/prednisolone to long-term hormone therapy in newly diagnosed advanced prostate cancer in England: Lifetime decision model based on STAMPEDE trial data
Source: PLoS One. 2022 Jun 2;17(6):e0269192. doi: 10.1371/journal.pone.0269192 (PMC9162346; doi:10.1371/journal.pone.0269192)
Supplement: S3 File — (PDF) [file pone.0269192.s004.pdf]

Cost-utility analysis of adding abiraterone acetate plus prednisone/prednisolone to long-term hormone therapy in newly diagnosed advanced prostate cancer: lifetime decision model in England based on STAMPEDE trial data

Caroline S Clarke*, Rachael M Hunter, Andrea Gabrio, Christopher D Brawley, Fiona C Ingleby, David P Dearnaley, David Matheson, Gerhardt Attard, Hannah L Rush, Rob J Jones, William Cross, Chris Parker, J Martin Russell, Robin Millman, Silke Gillessen, Zafar Malik, Jason F Lester, James Wylie, Noel W Clarke, Mahesh KB Parmar, Matthew R Sydes, Nicholas D James on behalf of the STAMPEDE investigators

* Corresponding author: caroline.clarke@ucl.ac.uk (CSC), Research Department of Primary Care and Population Health, University College London, London, UK.

Supporting Information

File 3

Relating to *Methods: Lifetime simulation model methods: Costs* section in main manuscript

Table S5 shows costs for active surveillance that were assumed for patients in HS1-3, based on Ramsay et al. [1] and adjusted to reflect STAMPEDE patients' characteristics according to clinical opinion, using the unit costs given in Table S6.

Table S5. Monitoring costs in earlier disease. Hormone-sensitive patients (HS1-3), costs for monitoring.

	Year 1	Year 2-5
Resource use	4 doctor-led outpatient appointments	2 doctor-led outpatient appointments
	4 PSA tests	2 PSA tests
	1 CT scan	2 CT scans
	1 bone scan	2 bone scans
	1 MDT meeting	-
Annual cost (£)	750	603
6-week cost (£)	86	69

Table S6. Unit costs for monitoring. These are the unit costs that feed into monitoring costs in Table S5 above.

Resource item	NHS Reference Cost code	Unit cost (£)	Source
Out-patient visit	Urology outpatient, service code 101	109.83	NHS Ref Costs 2017-18
CT scan	RD22Z, RD23Z, RD24Z, RD25Z, RD26Z, RD27Z	112.09	NHS Ref Costs 2017-18
Radiographic/MRI scan	RD03Z, RD04Z, RD05Z, RD06Z	174.05	NHS Ref Costs 2017-18
Bone scan	HRG RD50Z	77.46	NHS Ref Costs 2017-18
ECG	HRG RD51A	97.18	NHS Ref Costs 2017-18
Full blood count	HRG DAPS08	2.83	NHS Ref Costs 2017-18
Liver function test	HRG DAPS03	1.97	NHS Ref Costs 2017-18
Kidney function test	HRG DAPS03	1.97	NHS Ref Costs 2017-18
PSA	HRG DAPS03	1.97	NHS Ref Costs 2017-18
Cancer MDT Meeting	CMDT_Oth	113.50	NHS Ref Costs 2017-18

Table S7 shows the estimated monitoring costs for patients (in any health state) who were receiving chemotherapy, abiraterone acetate plus prednisone/prednisolone (AAP), or enzalutamide; and for those patients in HS4-7. In patients that were both in HS4-7 and receiving medications from this group where monitoring was more frequent, only the higher cost was applied.

Table S7. Monitoring costs in later disease. Monitoring costs for those patients receiving chemotherapy, abiraterone acetate plus prednisone/prednisolone (AAP), or enzalutamide, and for those in HS4-7.

	Receiving chemotherapy, AAP or enzalutamide		Castration-resistant prostate cancer (CRPC) patients	
	% of patients requiring	Frequency in a 3-month period	% of patients requiring	Frequency in a 3-month period
**Out-patient visit**	100	4	100	3
**CT scan**	80	1	60	1
**Radiographic /MRI scan**	5	1	10	1
**Bone scan**	50	1	50	1
**ECG**	10	1	10	1
**Full blood count**	100	4	100	3
**Liver function test**	100	4	100	3
**Kidney function test**	100	4	100	3
**PSA**	100	4	100	3
**Annual cost**	£2,485		£1,955	
**Total cost for 3 months**	£621		£489	
**Total cost for 6 weeks**	£286		£225	

Monitoring costs

Standard monitoring activities were not captured during STAMPEDE because patients were monitored via trial visits, so real-world costs of these were estimated as a flat rate for use in the lifetime simulation model via published information and clinical opinion, according to standard requirements should this intervention be implemented in England. These costs were split according to anticipated visit frequencies and costs: (i) patients receiving chemotherapy, AAP or enzalutamide, (ii) patients with naïve disease, not receiving chemotherapy, AAP or enzalutamide (split as year 1, years 2-5, then zero thereafter), and (iii) patients with CRPC disease, not receiving chemotherapy, AAP or enzalutamide. Monitoring costs for group (i) were included as an average amount within the daily medication costs for those medications, so a monitoring premium was included separately only for patients in groups (ii) and (iii). Additional monitoring costs for those not receiving any chemotherapy, AAP or enzalutamide were applied as a daily flat cost, of £2.09/day in hormone-naïve patients in year 1 of treatment and £1.65/day in years 2-5 of treatment, and £5.39/day in CRPC patients in any year of treatment.

References

[1] C. R. Ramsay, T. E. Adewuyi, J. Gray, J. Hislop, M. D. Shirley, S. Jayakody, G. MacLennan, C. Fraser, S. MacLennan, M. Brazzelli, J. N'Dow, R. Pickard, C. Robertson, K. Rothnie, S. P. Rushton, L. Vale and T. B. Lam, “Ablative therapy for people with localised prostate cancer: a systematic review and economic evaluation,” *Health Technology Assessment,* vol. 19, no. 49, 2015.
