# Supplementary material for: Cost-utility analysis of adding abiraterone acetate plus prednisone/prednisolone to long-term hormone therapy in newly diagnosed advanced prostate cancer in England: Lifetime decision model based on STAMPEDE trial data
Source: PLoS One. 2022 Jun 2;17(6):e0269192. doi: 10.1371/journal.pone.0269192 (PMC9162346; doi:10.1371/journal.pone.0269192)
Supplement: S4 File — (PDF) [file pone.0269192.s005.pdf]

Cost-utility analysis of adding abiraterone acetate plus prednisone/prednisolone to long-term hormone therapy in newly diagnosed advanced prostate cancer: lifetime decision model in England based on STAMPEDE trial data

Caroline S Clarke*, Rachael M Hunter, Andrea Gabrio, Christopher D Brawley, Fiona C Ingleby, David P Dearnaley, David Matheson, Gerhardt Attard, Hannah L Rush, Rob J Jones, William Cross, Chris Parker, J Martin Russell, Robin Millman, Silke Gillessen, Zafar Malik, Jason F Lester, James Wylie, Noel W Clarke, Mahesh KB Parmar, Matthew R Sydes, Nicholas D James on behalf of the STAMPEDE investigators

* Corresponding author: caroline.clarke@ucl.ac.uk (CSC), Research Department of Primary Care and Population Health, University College London, London, UK.

Supporting Information

File 4

Relating to *Results: Trial-based results: Trial population* section in the main manuscript

Table S8 shows the median, range and inter-quartile range follow-up times for patients in this trial analysis, split by subgroup and arm.

Table S8. Median, range and inter-quartile range follow-up times for patients in this trial analysis, split by subgroup and arm.

Follow-up (years)	n	Minimum	25% percentile	Median	75% percentile	Maximum
**M0 subgroup**	1011	0.02	2.95	3.08	3.57	5.03
AAP+SOC	515	0.02	2.96	3.08	3.60	5.03
SOC only	496	0.03	2.91	3.09	3.56	5.03
**M1 subgroup**	906	0.15	2.15	3.00	3.50	5.10
AAP+SOC	445	0.15	2.46	3.00	3.54	5.10
SOC only	461	0.20	1.94	2.92	3.47	5.10

Relating to *Results: Trial-based results: Survival modelling* section in the main manuscript

Table S9 shows the labels for the 25 transitions observed during the trial period. There were 9 models required in total: 6 for individual transitions and 3 for groups of jointly modelled transitions. The shaded groups indicate the three jointly modelled transitions: time to first event (i.e. transitions from HS1-3 to HS4/5/7/8), time to other-cause death (from HS1-7 to HS9), and transitions to the worst CRPC state (HS7) from the other three CRPC states (HS4-6), which was performed as a joint model due to small event numbers. Heavy borders indicate the six singly modelled transitions ([13, 15, 17, 19, 22, 24]).

Table S9. Labelling for the 25 transitions observed during the trial period.

from \ to	HS1	HS2	HS3	HS4	HS5	HS6	HS7	HS8	HS9
HS1				[1]	[2]		[3]	[4]	[5]
HS2					[6]		[7]	[8]	[9]
HS3							[10]	[11]	[12]
HS4					[13]		[14	[15]	[16]
HS5						[17]	[18]	[19]	[20]
HS6							[21]	[22]	[23]
HS7								[24]	[25]
HS8									
HS9									

The results of the model describing the time to first event, or treatment failure, are in Table S10 and show that those in the AAP+SOC arm had a significantly longer time to first event than the SOC-only arm. The values in the effect size columns in this table and the other tables below showing results for the other models were calculated as one over the exponential of the covariate effects, to improve interpretation. Larger effect size means longer time to survival event, i.e. better for the patient.

Table S10. Parameters of the joint survival model for failure-free survival using trial data, fitted using parametric splines with 5 degrees of freedom. *Significant at the 5% level. PSA = prostate-specific antigen level; Quintile 5 has worst disease, and Quintile 1 the least bad.

	Effect size	L95% CI	U95% CI
Control arm (A)	1.00		
Abiraterone arm (G)	18.20*	1.26	261.78
Mets at baseline: M0/M1a	1.00		
M1b (bone) or M1c (visceral)	0.33*	0.25	0.42
No lymph mets at baseline	1.00		
Lymph mets at baseline	0.79	0.62	1.00
No visceral mets at baseline	1.00		
Visceral mets at baseline	1.27	0.78	2.06
PSA Quintile 1 at baseline	1.00		
PSA Quintile 2 at baseline	0.68*	0.49	0.93
PSA Quintile 3 at baseline	0.87	0.62	1.20
PSA Quintile 4 at baseline	0.58*	0.43	0.79
PSA Quintile 5 at baseline	0.48*	0.35	0.65
RT not planned at baseline	1.00		
RT planned at baseline	2.98*	2.21	4.00
First transition 1, 6, 10 (naïve -> CRPC, same mets)	1.00		
First transition 2, 3, 7 (naïve -> CRPC, worse mets)	35.64*	25.10	50.58
Other-cause death	1.00		
Prostate cancer death	188.68*	83.82	425.53
Aged <60 at baseline	1.00		
Aged 60-64 at baseline	1.54*	1.14	2.09
Aged 65-69 at baseline	1.66*	1.26	2.20
Aged >70 at baseline	1.96*	1.49	2.57

The model parameters for time to other causes of death are in **Error! Reference source not found.**Table S11. Time to other-cause death depended on age, with the oldest patients having longer time to other-cause death compared to the youngest age group (an apparently contradictory but long-established finding [1]). The fit was improved when the four binary variables marking CRPC starting states were included.

Table S11. Parameters of the joint survival model for other-cause death using trial data, fitted using a log-normal model. *Significant at the 5% level.

	**Effect size**	**L95% CI**	**U95% CI**
Control arm (A)	1.00		
Abiraterone arm (G)	1.19	0.87	1.64
Aged <60 at baseline	1.00		
Aged 60-64 at baseline	1.37	0.75	2.52
Aged 65-69 at baseline	1.46	0.83	2.57
Aged >70 at baseline	2.20*	1.28	3.78
Not from HS4	1.00		
Transition from HS4	2.32*	1.30	4.12
Not from HS5	1.00		
Transition from HS5	2.13*	1.42	3.20
Not from HS6	1.00		
Transition from HS6	1.29	0.39	4.33
Not from HS7	1.00		
Transition from HS7	7.17*	3.72	13.79

Parameters for the jointly modelled transitions to the worst CRPC state (HS7) from the other CRPC states (HS4-6) are shown in Table S12. Those in the AAP+SOC arm had longer time to event than those in the SOC-only arm. Those transitioning from the worst preceding state (HS6) had shorter time to event (three-category variable describing the starting state for this transition), and older patients had shorter time to event.

Table S12. Parameters of the joint survival model describing the transitions to the most severe CRPC state (HS7) from the other three CRPC states (HS4-6) using trial data, fitted using a gamma model. *Significant at the 5% level.

	Effect size	L95% CI	U95% CI
SOC-only arm	1.00		
AAP+SOC arm	1.63	0.69	3.85
PSA Quintile 1 at baseline	1.00		
PSA Quintile 2 at baseline	2.17	0.62	7.58
PSA Quintile 3 at baseline	0.44	0.12	1.60
PSA Quintile 4 at baseline	0.28	0.05	1.46
PSA Quintile 5 at baseline	0.74	0.18	2.98
Aged <60 at baseline	1.00		
Aged 60-64 at baseline	0.26*	0.08	0.80
Aged 65-69 at baseline	0.58	0.20	1.69
Aged >70 at baseline	0.12*	0.04	0.35
Transition from HS4	1.00		
Transition from HS5	0.68	0.22	2.11
Transition from HS6	0.08*	0.02	0.34

The values in the effect size columns in Tables S12 to S18 inclusive were calculated as one over the exponential of the covariate effects, to improve interpretation. The six remaining singly modelled transitions also used the randomisation variables and a variety of other relevant covariates, and all consistently showed that being in the AAP+SOC arm allowed a longer time to event than being in the SOC-only arm, with the inclusion of this covariate being statistically significant for transitions 15, 17 and 19, and not statistically significant for 13, 22 and 24. Table S13 shows that those in AAP+SOC arm had longer to event although it is not a significant difference. Table S14 shows that those in AAP+SOC arm had significantly longer to event; those with baseline lymph node metastases had significantly longer time to event (vs. those with no lymph node metastases). Including the interaction term between treatment arm and lymph node metastatic status improved the model fit. Table S15 shows that those in AAP+SOC arm had significantly longer to event; those with worse (higher) baseline Gleason scores had significantly longer to event. Including the variable indicating those recruited when arm H was open improved the model fit. Table S16 shows that those in AAP+SOC arm had significantly longer to event; those with worse (higher) baseline WHO performance status (PS) had significantly longer to event. Including the interaction term between treatment arm and WHO PS improved the fit and was statistically significant. Table S17 shows that those with worse (higher) baseline WHO PS had significantly shorter to event. Including the randomisation

variable and the variable indicating that arm H had opened improved the model fit. Table S18 shows that those with baseline visceral metastases had shorter time to first event, compared to those with either bone, lymph node or no metastases. Including the randomisation variable and the variable indicating that arm H had opened improved the model fit.

Table S13. Parameters of the survival model for the single transition from HS4 to HS5 using trial data, fitted using a gamma model.

T13: HS4 to HS5	Effect size	L95% CI	U95% CI
SOC-only arm	1.00		
AAP+SOC arm	1.48	0.35	6.21

Table S14. Parameters of the survival model for the single transition from HS4 to HS8 using trial data, fitted using a Weibull model. *Significant at the 5% level.

T15: HS4 to HS8	Effect size	L95% CI	U95% CI
SOC-only arm	1.00		
AAP+SOC arm	7.14*	2.27	22.42
No lymph mets at baseline	1.00		
Lymph mets at baseline	6.27*	1.97	19.96
Lymph mets at baseline: SOC-only arm	1.00		
Lymph mets at baseline: AAP+SOC arm (interaction effect)	0.30	0.08	1.13

Table S15. Parameters of the survival model for the single transition from HS5 to HS6 using trial data, fitted using a Weibull model. *Significant at the 5% level.

T17: HS5 to HS6	Effect size	L95% CI	U95% CI
SOC-only arm	1.00		
AAP+SOC arm	2.38*	1.52	3.73
Gleason score ≤7 at baseline	1.00		
Gleason score ≥8 at baseline	2.07*	1.07	4.00
Gleason score unknown at baseline	1.62	0.35	7.45
Arm H not yet open	1.00		
Arm H open at recruitment	1.55	0.99	2.42

Table S16. Parameters of the survival model for the single transition from HS5 to HS8 using trial data, fitted using a Weibull model. *Significant at the 5% level.

T19: HS5 to HS8	Effect size	L95% CI	U95% CI
SOC-only arm	1.00		
AAP+SOC arm	1.81*	1.42	2.31
WHO grade 0 at baseline	1.00		
WHO grade 1 or 2 at baseline	1.47*	1.13	1.90
WHO grade 1 or 2: SOC-only arm	1.00		
WHO grade 1 or 2: AAP+SOC arm (interaction effect)	0.62*	0.38	0.99

Table S17. Parameters of the survival model for the single transition from HS6 to HS8 using trial data, fitted using a Weibull model. *Significant at the 5% level.

T22: HS6 to HS8	Effect size	L95% CI	U95% CI
SOC-only arm	1.00		
AAP+SOC arm	1.02	0.71	1.47
WHO PS 0 at baseline	1.00		
WHO PS 1 or 2 at baseline	0.63*	0.42	0.92
Arm H not yet open	1.00		
Arm H open at recruitment	0.75	0.53	1.06

Table S18. Parameters of the survival model for the single transition from HS7 to HS8 using trial data, fitted using a log-normal model. *Significant at the 5% level.

T24: HS7 to HS8	Effect size	L95% CI	U95% CI
SOC-only arm	1.00		
AAP+SOC arm	1.33	0.81	2.17
No visceral mets at baseline	1.00		
Visceral mets at baseline	0.34*	0.21	0.56

Validation of survival models

The AAP+ADT arm in the LATITUDE trial showed comparable survival to 66 months compared to the AAP+SOC arm in STAMPEDE abiraterone comparison [2]; the AAP arm in the COU-AA-301 and COU-AA-302 studies in CRPC patients with bone metastases had shorter follow-up (25 months) but also agreed with the present model over the available timeframe [3]; the prednisolone (control) arm in COMET-1 also had a shorter follow-up (median 21 months), but broadly agreed over this timeframe [4]; and the AAP plus placebo arm in ERA 223, looking at addition of radium-223 to AAP gave similar agreement over a 43-month timeframe [5]. Analysis was performed in R (version 3.6.3) [6] with some preparatory work performed in Stata v14 [7] and v16 [8].

References

[1] A. Stangelberger, M. Waldert and B. Djavan, “Prostate Cancer in Elderly Men,” *Reviews in Urology,* vol. 10, no. 2, pp. 111-119, 2008.

[2] K. Fizazi, N. Tran, L. Fein, N. Matsubara, A. Rodriguez-Antolin, B. Y. Alekseev, M. Özgüroğlu, D. Ye, S. Feyerabend, A. Protheroe, P. De Porre, T. Kheoh, Y. C. Park, M. B. Todd and K. N. Chi, “Abiraterone plus Prednisone in Metastatic, Castration-Sensitive Prostate Cancer,” *New England Journal of Medicine,* vol. 377, pp. 352-360, 2017.

[3] C. J. Ryan, M. R. Smith, K. Fizazi, F. Saad, P. F. Mulders, C. N. Sternberg, K. Miller, C. J. Logothetis, N. D. Shore, E. J. Small, J. Carles, T. W. Flaig, M.-E. Taplin, C. S. Higano, P. de Souza, J. S. de Bono, T. W. Griffin, P. De Porre, M. K. Yu, Y. C. Park, J. Li, T. Kheoh, V. Naini, A. Molina and D. E. Rathkopf, “Abiraterone acetate plus prednisone versus placebo plus prednisone in chemotherapy-naive men with metastatic castration-resistant prostate cancer (COU-AA-302): final overall survival analysis of a randomised, double-blind, placebo-controlled phase 3 study,” *Lancet Oncology,* vol. 16, pp. 152-160, 2015.

[4] M. Smith, J. De Bono, C. Sternberg, S. Le Moulec, S. Oudard, U. De Giorgi, M. Krainer, A. Bergman, W. Hoelzer, R. De Wit, M. Bogemann, F. Saad, G. Cruciani, A. Thiery-Vuillemin, S. Feyerabend, K. Miller, N. Houede, S. Hussain, E. Lam, J. Polikoff, A. Stenzl, P. Mainwaring, D. Ramies, C. Hessel, A. Weitzman and K. Fizazi, “Phase III Study of Cabozantinib in Previously Treated Metastatic Castration-Resistant Prostate Cancer: COMET-1,” *Journal of Clinical Oncology,* vol. 34, no. 25, pp. 3005-3013, 2016.

[5] M. Smith, C. Parker, F. Saad, K. Miller, B. Tombal, Q. S. Ng, M. Boegemann, V. Matveev, J. M. Piulats, L. E. Zucca, O. Karyakin, G. Kimura, N. Matsubara, W. C. Nahas, F. Nole, E. Rosenbaum, A. Heidenreich, Y. Kakehi, A. Zhang, H. Krissel, M. Teufel, J. Shen, V. Wagner and C. Higano, “Addition of radium-223 to abiraterone acetate and prednisone or prednisolone in patients with castration-resistant prostate cancer and bone metastases (ERA 223): a randomised, double-blind, placebo-controlled, phase 3 trial,” *Lancet Oncology,* vol. 20, pp. 408-419, 2019.

[6] R Core Team, *R: A language and environment for statistical computing,* Vienna: R Foundation for Statistical Computing, 2020.

[7] StataCorp, *Stata Statistical Software: Release 14,* College Station, TX, USA: StataCorp LP, 2015.

[8] StataCorp, *Stata Statistical Software: Release 16,* College Station, TX, USA: StataCorp LP, 2019.
