# Supplementary material for: Cost-utility analysis of adding abiraterone acetate plus prednisone/prednisolone to long-term hormone therapy in newly diagnosed advanced prostate cancer in England: Lifetime decision model based on STAMPEDE trial data
Source: PLoS One. 2022 Jun 2;17(6):e0269192. doi: 10.1371/journal.pone.0269192 (PMC9162346; doi:10.1371/journal.pone.0269192)
Supplement: S5 File — (PDF) [file pone.0269192.s006.pdf]

Cost-utility analysis of adding abiraterone acetate plus prednisone/prednisolone to long-term hormone therapy in newly diagnosed advanced prostate cancer: lifetime decision model in England based on STAMPEDE trial data

Caroline S Clarke*, Rachael M Hunter, Andrea Gabrio, Christopher D Brawley, Fiona C Ingleby, David P Dearnaley, David Matheson, Gerhardt Attard, Hannah L Rush, Rob J Jones, William Cross, Chris Parker, J Martin Russell, Robin Millman, Silke Gillessen, Zafar Malik, Jason F Lester, James Wylie, Noel W Clarke, Mahesh KB Parmar, Matthew R Sydes, Nicholas D James on behalf of the STAMPEDE investigators

* Corresponding author: caroline.clarke@ucl.ac.uk (CSC), Research Department of Primary Care and Population Health, University College London, London, UK.

Supporting Information

File 5

Relating to *Results: Trial-based results: Utilities* section in the main manuscript

Fig S1 shows how the mean, unimputed utility score changed over time in each arm. Sample size at later timepoints was small, partly due to data not being routinely collected after progression, hence the apparent fluctuations from 182 weeks onwards. Despite the protocol not requiring EQ-5D-3L to be collected after progression, some patients did report this information, allowing utility scores to be modelled using only trial data.

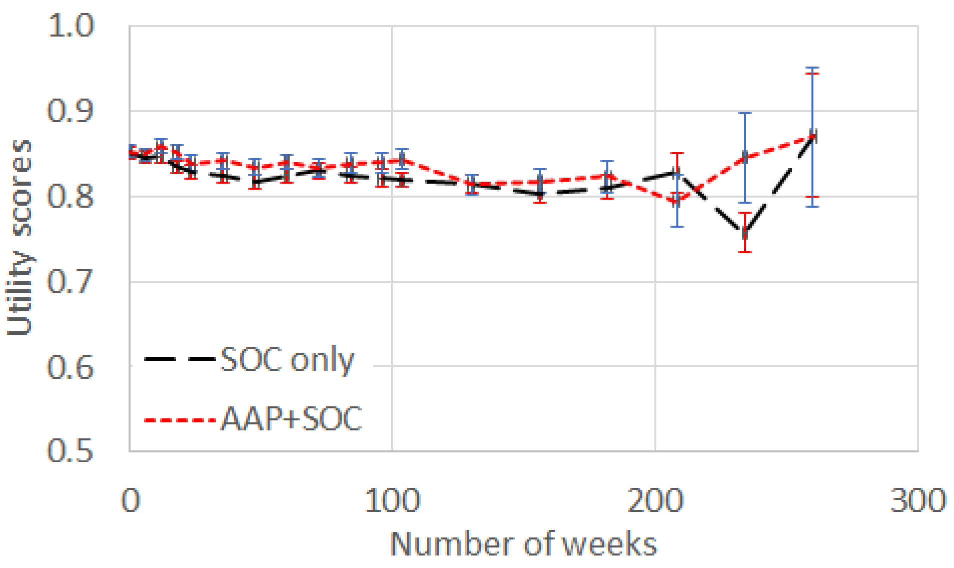

Weeks	0	6	12	18	24	36	48	60	72	84	96	104	130	156	182	208	234	260
AAP+SOC	647	633	625	582	631	603	598	585	563	529	491	532	494	421	245	128	49	12
SOC-only	628	653	598	568	594	599	573	522	493	463	405	444	412	324	172	76	39	10

Fig S1. Mean undiscounted utility scores per arm and timepoint for alive patients; the table provides information on the sample size at each timepoint used to calculate these scores. Short-dotted (red) line is AAP+SOC, and long-dashed (black) line is SOC-only. Responses were assigned to the closest listed week according to the date on which the data were collected.

Table S19. Unimputed trial utility scores by arm and health state. HS1 and HS4 contain only M0 subgroup patients; HS2 and HS3 contain only M1 subgroup patients; and HS5-7 contain a mixture of the two subgroups.

Utility scores	AAP+SOC arm			SOC-only arm		
	n	Mean	SD	n	Mean	SD
**HS1**	4595	0.846	0.203	3786	0.857	0.202
**HS2**	3140	0.846	0.193	2097	0.832	0.214
**HS3**	270	0.771	0.289	206	0.823	0.212
**HS4**	49	0.758	0.249	450	0.821	0.228
**HS5**	260	0.761	0.220	903	0.739	0.266
**HS6**	29	0.693	0.324	63	0.655	0.274
**HS7**	25	0.783	0.277	68	0.645	0.304

Table S20. Utility scores and missingness patterns in trial data in AAP+SOC arm.

	Numbers of patients			Unimputed trial utility scores			
	Total	Dead	Missing	Mean	Std. Dev.	Min	Max
All timepoints	898	0	0	0.839	0.205	-0.248	1
0 weeks	647	0	251	0.853	0.181	-0.181	1
6 weeks	633	0	265	0.849	0.198	-0.184	1
12 weeks	625	1	272	0.858	0.190	-0.239	1
18 weeks	582	3	313	0.853	0.180	-0.074	1
24 weeks	631	5	262	0.838	0.207	-0.239	1
36 weeks	603	8	287	0.842	0.206	-0.239	1
48 weeks	598	18	282	0.834	0.203	-0.077	1
60 weeks	585	31	282	0.841	0.206	-0.239	1
72 weeks	563	42	293	0.833	0.220	-0.239	1
84 weeks	529	67	302	0.839	0.201	-0.248	1
96 weeks	491	77	330	0.840	0.211	-0.135	1
104 weeks	532	89	277	0.843	0.201	-0.239	1
130 weeks	494	121	283	0.814	0.232	-0.239	1
156 weeks	421	155	322	0.818	0.225	-0.239	1
182 weeks	245	169	484	0.823	0.221	-0.239	1
208 weeks	128	181	589	0.794	0.263	-0.074	1
234 weeks	49	183	666	0.845	0.164	0.487	1
260 weeks	12	184	702	0.870	0.249	0.189	1

Table S21. Utility scores and missingness patterns in trial data in SOC-only arm.

	Numbers of patients			Unimputed trial utility scores			
	Total	Dead	Missing	Mean	Std. Dev.	Min	Max
All timepoints	896	0	0	0.829	0.222	-0.429	1
0 weeks	628	0	268	0.851	0.191	-0.181	1
6 weeks	653	0	243	0.846	0.194	-0.239	1
12 weeks	598	3	295	0.847	0.197	-0.181	1
18 weeks	568	7	321	0.835	0.201	-0.074	1
24 weeks	594	12	290	0.829	0.231	-0.181	1
36 weeks	599	23	274	0.825	0.226	-0.239	1
48 weeks	573	35	288	0.817	0.224	-0.349	1
60 weeks	522	45	329	0.824	0.207	-0.074	1
72 weeks	493	65	338	0.830	0.223	-0.239	1
84 weeks	463	87	346	0.825	0.231	-0.181	1
96 weeks	405	107	384	0.822	0.232	-0.239	1
104 weeks	444	125	327	0.819	0.245	-0.239	1
130 weeks	412	159	325	0.815	0.251	-0.429	1
156 weeks	324	217	355	0.804	0.249	-0.239	1
182 weeks	172	238	486	0.810	0.252	-0.181	1
208 weeks	76	253	567	0.828	0.263	-0.349	1
234 weeks	39	261	596	0.757	0.324	-0.170	1
260 weeks	10	262	624	0.871	0.260	0.189	1

Table S22. Regression parameters from two-part regression of utility scores using trial data.

	Odds ratio from first part of regression model	QOL decrement from second part of regression model
	**Mean (95% CI)**	**Mean (95% CI)**
**Constant (odds)**	0.41 (0.36, 0.46)*	0.43 (0.30, 0.56)*
**WHO status (ref. cat. = 0)**		
1 and 2	0.72 (0.66, 0.78)*	0.07 (0.05, 0.08)*
**Age (ref. cat. = ≤60 years)**		
60-64	1.13 (1.06, 1.21)*	-0.02 (-0.04, 0.00)
65-69	1.31 (1.21, 1.42)*	-0.05 (-0.07, -0.03)*
≥70	1.30 (1.21, 1.40)*	-0.06 (-0.08, -0.04)*
**Nodal status (ref. cat. = N0)**		
N+	0.97 (0.93, 1.00)	0.00 (-0.01, 0.02)
NX (unknown)	1.03 (0.94, 1.12)	0.00 (-0.03, 0.03)
**Treatment (ref. cat. = second year onwards)**		
First year on SOC	0.99 (0.74, 1.32)	-0.06 (-0.21, 0.09)
First year on AAP+SOC	1.03 (0.86, 1.25)	-0.10 (-0.28, 0.09)
**Health state (ref. cat. HS1)**		
HS2	0.96 (0.92, 0.99)*	-0.01 (-0.02, 0.01)
HS3	0.93 (0.81, 1.06)	-0.01 (-0.06, 0.04)
HS4	0.90 (0.80, 1.02)	0.05 (-0.01, 0.12)
HS5	0.70 (0.59, 0.84)*	0.06 (0.02, 0.10)*
HS6	0.63 (0.38, 1.04)	0.10 (0.02, 0.18)*
HS7	0.54 (0.39, 0.74)*	0.10 (0.05, 0.15)*

* indicates statistical significance at the 5% level

Deterministic sensitivity analysis of trial utilities

Fig S2 shows a Tornado diagram of the estimated mean (95% CI) for the combined coefficients of the two-part regression model, generating using bootstrapping, to explore how baseline covariates predict quality of life. The reference categories are: node negative, younger than 60 years, WHO performance status 0, hormone naïve M0, and EQ-5D-3L collected more than a year after randomisation. This analysis suggests that older patients have better quality of life, which agrees with published work suggesting that patients who are older at diagnosis (and therefore at entry into STAMPEDE) have less severe disease. Other disease severity markers, including health states and WHO performance status, show that worse disease coincides with lower utility score, thus validating the model. The three-category “treatment in first year” parameter result here suggests that utility could be slightly better in AAP+SOC arm in the first year, and SOC-only arm in the first year, although this inference is not statistically significant due to wide confidence intervals.

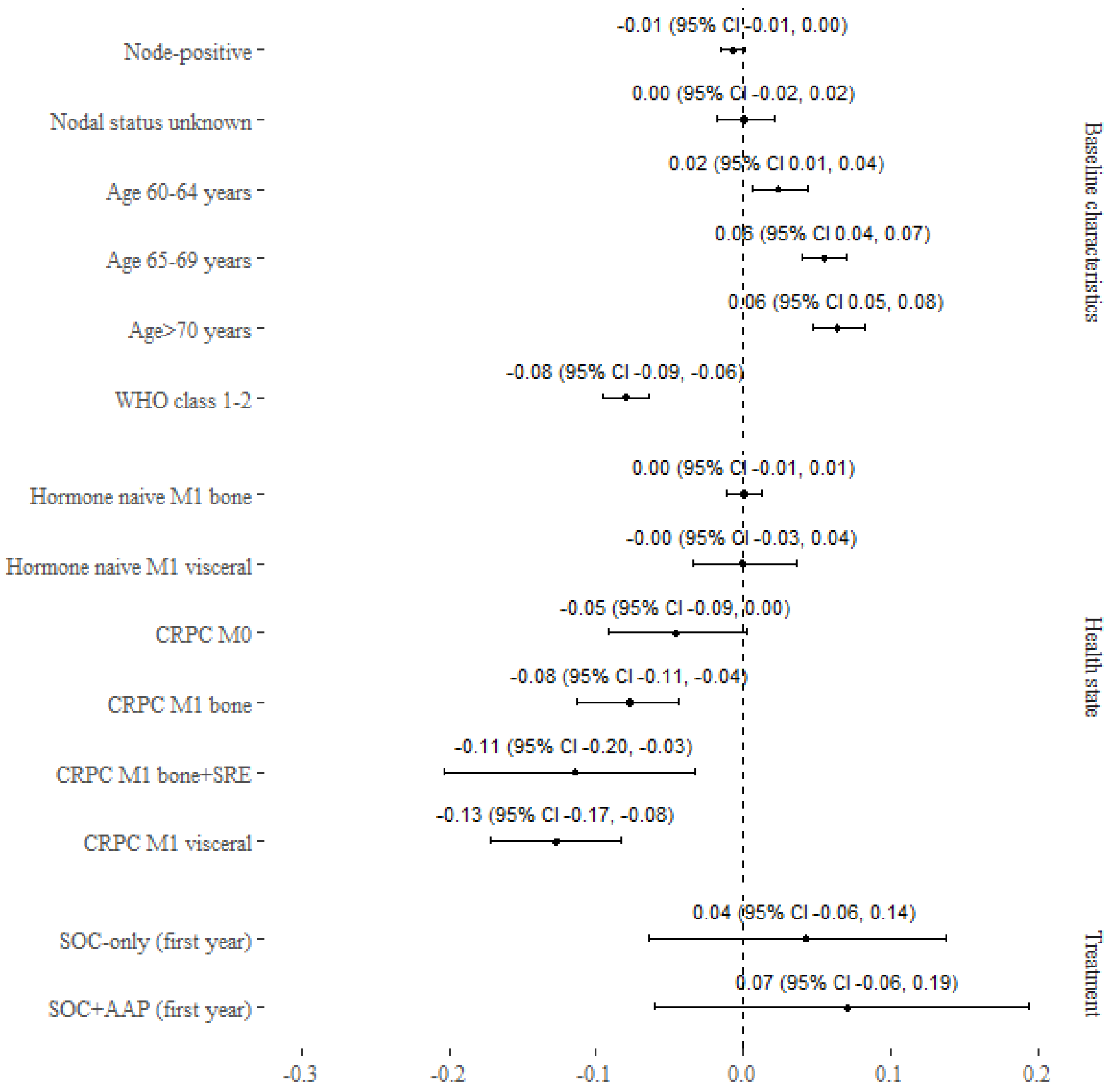

Fig S2: Deterministic sensitivity analysis. Impact of baseline characteristics, health state and treatment allocation on patient health-related quality of life scores in STAMPEDE.

Table S23. Imputed trial utility data. Data from the imputation that was used in the two-part regression are in the left-hand half of this table, and values predicted via this regression are presented in the right-hand half of this table.

Utility scores	Imputed trial data (one imputation)				Predicted using two-part regression of trial data			
	AAP+SOC		SOC-only		AAP+SOC		SOC-only	
	Mean	SD	Mean	SD	Mean	SD	Mean	SD
**HS1**	0.782	0.297	0.779	0.311	0.764	0.057	0.801	0.040
**HS2**	0.780	0.290	0.756	0.315	0.754	0.059	0.796	0.042
**HS3**	0.748	0.307	0.820	0.233	0.770	0.060	0.802	0.044
**HS4**	0.752	0.303	0.742	0.329	0.716	0.064	0.744	0.036
**HS5**	0.771	0.259	0.673	0.350	0.685	0.054	0.698	0.045
**HS6**	0.745	0.298	0.604	0.371	0.668	0.044	0.650	0.052
**HS7**	0.759	0.292	0.598	0.364	0.632	0.060	0.654	0.036
