# Supplementary material for: Cost-utility analysis of adding abiraterone acetate plus prednisone/prednisolone to long-term hormone therapy in newly diagnosed advanced prostate cancer in England: Lifetime decision model based on STAMPEDE trial data
Source: PLoS One. 2022 Jun 2;17(6):e0269192. doi: 10.1371/journal.pone.0269192 (PMC9162346; doi:10.1371/journal.pone.0269192)
Supplement: S6 File — (PDF) [file pone.0269192.s007.pdf]

Cost-utility analysis of adding abiraterone acetate plus prednisone/prednisolone to long-term hormone therapy in newly diagnosed advanced prostate cancer: lifetime decision model in England based on STAMPEDE trial data

Caroline S Clarke*, Rachael M Hunter, Andrea Gabrio, Christopher D Brawley, Fiona C Ingleby, David P Dearnaley, David Matheson, Gerhardt Attard, Hannah L Rush, Rob J Jones, William Cross, Chris Parker, J Martin Russell, Robin Millman, Silke Gillessen, Zafar Malik, Jason F Lester, James Wylie, Noel W Clarke, Mahesh KB Parmar, Matthew R Sydes, Nicholas D James on behalf of the STAMPEDE investigators

* Corresponding author: caroline.clarke@ucl.ac.uk (CSC), Research Department of Primary Care and Population Health, University College London, London, UK.

Supporting Information

File 6

Relating to *Results: Trial-based results: Cost information from trial data* section in the main manuscript.

Mean daily per-patient costs of other therapies and medications including ADT, dexamethasone, chemotherapy, radioisotopes, bisphosphonates and specified other medications are given in Table S24.

Table S24. Mean daily undiscounted per-patient costs of other medications: raw reported doses and imputed doses according the BNF and modal observed doses where available.

	Number pts	Daily* cost of raw reported dose			Daily* cost of imputed dose			Treatment duration* (days) (to censor date or earlier)	
**Hormone Therapy (except enza)**	**n pts**	**n events**	**mean (£)**	**SD (£)**	**n events**	**mean (£)**	**SD (£)**	**mean**	**SD**
AAP+SOC arm	951	1822	6.51	37.24	1985	6.27	35.68	941.1	479.8
SOC-only arm	951	1859	6.76	39.66	2409	6.12	34.86	771.2	495.2
**Steroids (dexamethasone)**									
AAP+SOC arm	53	34	0.49	0.20	64	0.43	0.16	178.7	209.8
SOC-only arm	117	98	0.54	0.41	152	0.47	0.34	187.8	206.2
**Chemotherapy (excluding docetaxel and cabazitaxel)**									
AAP+SOC arm	7	0			11	68.83	39.77	132.5	94.8
SOC-only arm	4	0			6	66.31	40.68	49.3	53.1
**Bisphosphonates**									
AAP+SOC arm	43	0			43	5.88	0.76	249.0	184.7
SOC-only arm	84	0			95	5.57	1.43	317.5	247.6
**Radioisotopes (Strontium-89)***									
AAP+SOC arm	0	0			0			n/a	n/a
SOC-only arm	2	0			2	2153	0	n/a	n/a
**Other medications (pembrolizumab and tamsulosin)**									
AAP+SOC arm	0	0			0				
SOC-only arm	2	0			2	125.26	177.08	209.0	48.1

*Total rather than daily cost is given here for Sr-89, as it was included as a one-off cost on the medication start date.

Information on radiotherapy administered during the trial was largely complete regarding numbers of fractions, doses and start and stop dates. A radiotherapy preparation cost was included at the start of each RT delivery series, and a delivery cost was applied at each fraction administration, according to NHS Reference Costs. Information reported during the trial is summarised in Table S25 as the total cost per person during the trial period. Modal doses or numbers of fractions were imputed where that information was missing. Costs for procedures performed during the trial are also shown in Table S25, and no information was imputed to calculate costs of procedures.

Table S25. Total undiscounted per-patient costs of radiotherapy and procedures during the period of time that the therapy was used during the trial.

	Number pts	Total per-person cost of reported dose			Total per-person cost of imputed dose		
	**n pts**	**n events**	**mean (£)**	**SD (£)**	**n events**	**mean (£)**	**SD (£)**
**Radiotherapy (curative)**							
AAP+SOC arm	449	480	5352.26	2129.23	486	5308.89	2152.04
SOC-only arm	536	627	4429.04	2429.02	642	4377.86	2431.18
**Radiotherapy (palliative)**							
AAP+SOC arm	30	41	1718.43	826.48	41	1718.43	826.48
SOC-only arm	78	114	1775.46	546.98	115	1772.21	545.70
**Procedures**							
AAP+SOC arm	79	122	2316.04	2057.52	122	2316.04	2057.52
SOC-only arm	91	129	2673.27	2433.13	129	2673.27	2433.13

Total costs per patient for unscheduled primary and secondary care visits during the trial are presented in Table S26.

Table S26. Undiscounted costs for unscheduled primary and secondary care visits during the trial.

	Number pts	Total per-person cost of reported visits (£)		
	**n pts**	**n events**	**mean**	**SD**
**Unscheduled primary/secondary care visits**				
AAP+SOC arm	512	1250	637.18	1377.48
SOC-only arm	533	1354	848.20	1822.56

Table S27 provided the estimated annual costs from the two-part regression model using the patient-level trial data.

Table S27. Estimated annual costs from two-part regression model for general disease management costs found using trial data, for the different baseline characteristics categories used in the regression.

	Annual costs from regression of costs below £1500 per cycle	Annual costs from regression of costs above £1500 per cycle
	**Mean (95% CI)**	**Mean (95% CI)**
**Constant**	1291 (215, 2367)*	1836 (601, 3071)*
**WHO status (ref. cat. = 0)**		
1 and 2	117 (-72, 306)	236 (-611, 1084)
**Age (ref. cat. = ≤60 years)**		
60-64	-335 (-1450, 780)	254 (-1160, 1669)
65-69	-790 (-1835, 255)	-395 (-1488, 697)
≥70	-788 (-1833, 258)	-650 (-1704, 404)
**Nodal status (ref. cat. = N0)**		
N+	332 (120, 543)*	-734 (-1444, -25)*
NX (unknown)	-344 (-737, 49)	-1117 (-1985, -250)*
**Treatment (ref. cat. = second year onwards)**		
First year on SOC	4184 (3387, 4981)*	4626 (3767, 5486)*
First year on SOC+Abi	3580 (3223, 3937)*	3740 (3237, 4244)*
**Health state (ref. cat. HS1)**		
HS2	1155 (708, 1602)*	-1235 (-1854, -615)*
HS3	461 (73, 850)*	-1021 (-3362, 1320)
HS4	562 (279, 844)*	1715 (1188, 2242)*
HS5	1355 (1013, 1696*)	1414 (748, 2080)*
HS6	247 (-244, 738)	-121 (-1731, 1489)
HS7	173 (-233, 578)	241 (-569, 1051)

Table S28 provides the reported numbers of events, with costs calculated from imputed doses as dose information was missing, and reported treatment duration, from the trial information.

Table S28. Mean daily undiscounted per-patient costs of docetaxel, enzalutamide, cabazitaxel and radium when reported for disease progression; no dose information was reported in the trial. Imputed doses according the BNF and modal observed doses where available.

	Daily* cost of imputed dose			Treatment duration* (days)	
	**n events**	**mean (£)**	**SD (£)**	**mean**	**SD**
**Docetaxel**					
AAP+SOC arm	113	57.21	0	176.0	135.2
SOC-only arm	203	57.21	0	207.0	206.0
**Enzalutamide**					
AAP+SOC arm	28	97.67	0	205.4	160.1
SOC-only arm	149	97.67	0	243.7	187.9
**Cabazitaxel**					
AAP+SOC arm	17	177.24	0	133.1	109.7
SOC-only arm	25	177.24	0	163.4	128.6
**Radioisotopes (Radium-223)***					
AAP+SOC arm	20	19392	0	n/a	n/a
SOC-only arm	22	19392	0	n/a	n/a

*Total rather than daily cost is given here for Ra-223, as it was included as a one-off cost on the medication start date.

References

[1] B. S. Woods, E. Sideris, M. R. Sydes, M. R. Gannon, M. K. Parmar, M. Alzouebi, G. Attard, A. J. Birtle and S. Brock, "Addition of Docetaxel to First-line Long-term Hormone Therapy in Prostate Cancer (STAMPEDE): Modelling to Estimate Long-term Survival, Quality-adjusted Survival, and Cost-effectiveness," *European Urology Oncology,* pp. 449-458, 2018.
