# Supplementary material for: Cost-utility analysis of adding abiraterone acetate plus prednisone/prednisolone to long-term hormone therapy in newly diagnosed advanced prostate cancer in England: Lifetime decision model based on STAMPEDE trial data
Source: PLoS One. 2022 Jun 2;17(6):e0269192. doi: 10.1371/journal.pone.0269192 (PMC9162346; doi:10.1371/journal.pone.0269192)
Supplement: S8 File — (PDF) [file pone.0269192.s009.pdf]

Cost-utility analysis of adding abiraterone acetate plus prednisone/prednisolone to long-term hormone therapy in newly diagnosed advanced prostate cancer: lifetime decision model in England based on STAMPEDE trial data

Caroline S Clarke*, Rachael M Hunter, Andrea Gabrio, Christopher D Brawley, Fiona C Ingleby, David P Dearnaley, David Matheson, Gerhardt Attard, Hannah L Rush, Rob J Jones, William Cross, Chris Parker, J Martin Russell, Robin Millman, Silke Gillessen, Zafar Malik, Jason F Lester, James Wylie, Noel W Clarke, Mahesh KB Parmar, Matthew R Sydes, Nicholas D James on behalf of the STAMPEDE investigators

* Corresponding author: caroline.clarke@ucl.ac.uk (CSC), Research Department of Primary Care and Population Health, University College London, London, UK.

Supporting Information

File 8

Relating to *Results: Headline results from overall lifetime model* section in the main manuscript

Table S29 shows utility scores estimated using the base case deterministic lifetime simulation, by health state, subgroup and arm. The lifetime QALY results from the base case deterministic lifetime simulation are summarised in Table S30 below. The base-case discounted lifetime costs are summarised in Table S31, split according to cost categories.

Table S29. Utility scores estimated by the lifetime simulation model, split by health state, subgroup and arm, with missing values imputed using MICE.

	HS1	HS2	HS3	HS4	HS5	HS6	HS7
M1 AAP+SOC	-	0.715	0.705	-	0.638	0.601	0.587
M1 SOC-only	-	0.715	0.705	-	0.638	0.601	0.588
M0 AAP+SOC	0.722	-	-	0.677	0.643	0.605	0.592
M0 SOC-only	0.722	-	-	0.677	0.646	0.609	0.596
AAP+SOC (all)	0.722	0.715	0.705	0.677	0.641	0.603	0.589
SOC-only (all)	0.722	0.715	0.705	0.677	0.643	0.605	0.592
All	0.722	0.715	0.705	0.677	0.642	0.604	0.591

Table S30. Estimated discounted lifetime mean QALYs, split by arm and subgroup, and grouped by hormone-naïve vs. CRPC health states, calculated using the lifetime simulation model.

	M0 subgroup			M1 subgroup		
	AAP+SOC	SOC-only	Difference	AAP+SOC	SOC-only	Difference
**QALYs (discounted):**						
Hormone-naïve health states (HS1-3)	6.72	4.72	2.01	3.79	1.65	2.14
CRPC health states (HS4-7)	0.31	1.99	-1.68	0.64	1.30	-0.66
**Total lifetime QALYs**	7.03	6.70	**0.33**	4.43	2.95	**1.48**

Table S31. Per-patient costs split by arm and subgroup, for the various cost categories used in the lifetime simulation model, calculated using the deterministic base case analysis.

	M0 subgroup			M1 subgroup		
	AAP+SOC	SOC-only	Difference	AAP+SOC	SOC-only	Difference
AAP incl. monitoring	69,573	10,302	59,271	91,862	16,971	74,892
Monitoring (except AAP)	2,923	4,395	-1,472	1,294	1,350	-56
Group of four specific medications	1,342	4,678	-3,336	5,134	9,660	-4,525
General disease management	21,985	26,952	-4,968	14,001	12,883	1,118
Serious adverse events (SAEs)	396	226	170	396	226	170
End of life care	1,340	2,184	-844	3,969	5,321	-1,352
**Total lifetime costs**	97,558	48,736	**48,821**	116,658	46,412	**70,246**
