# Supplementary material for: Cost-utility analysis of adding abiraterone acetate plus prednisone/prednisolone to long-term hormone therapy in newly diagnosed advanced prostate cancer in England: Lifetime decision model based on STAMPEDE trial data
Source: PLoS One. 2022 Jun 2;17(6):e0269192. doi: 10.1371/journal.pone.0269192 (PMC9162346; doi:10.1371/journal.pone.0269192)
Supplement: S9 File — (PDF) [file pone.0269192.s010.pdf]

Cost-utility analysis of adding abiraterone acetate plus prednisone/prednisolone to long-term hormone therapy in newly diagnosed advanced prostate cancer: lifetime decision model in England based on STAMPEDE trial data

Caroline S Clarke*, Rachael M Hunter, Andrea Gabrio, Christopher D Brawley, Fiona C Ingleby, David P Dearnaley, David Matheson, Gerhardt Attard, Hannah L Rush, Rob J Jones, William Cross, Chris Parker, J Martin Russell, Robin Millman, Silke Gillessen, Zafar Malik, Jason F Lester, James Wylie, Noel W Clarke, Mahesh KB Parmar, Matthew R Sydes, Nicholas D James on behalf of the STAMPEDE investigators

* Corresponding author: caroline.clarke@ucl.ac.uk (CSC), Research Department of Primary Care and Population Health, University College London, London, UK.

Supporting Information

File 9

Relating to *Results: Lifetime simulation model results: Probabilistic sensitivity analysis* section in the main manuscript

Figs S4 and S5 show the results of the probabilistic sensitivity analysis for the M0 subgroup.

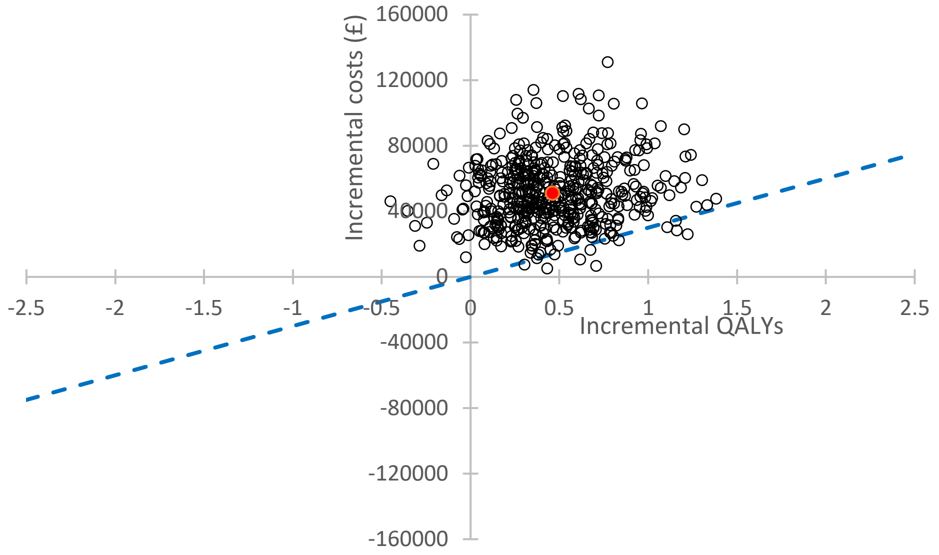

Fig S4. Cost-effectiveness plane for M0 subgroup. This used the base case 100% BNF price for abiraterone, showing that AAP+SOC was both more expensive and more effective than SOC-only (i.e. points found in north-east quadrant) the majority of the time. The red point indicates the mean incremental cost plotted against the mean incremental QALYs in this set of probabilistic results, and the blue dashed line indicates the £30,000/QALY gained threshold.

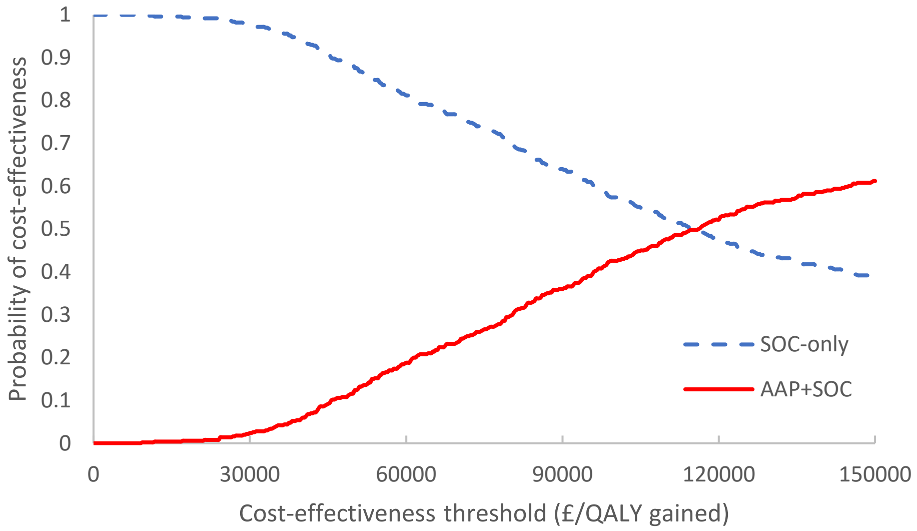

Fig S5. Cost-effectiveness acceptability curve (CEAC) for M0 subgroup. This used the base case 100% BNF price for abiraterone, and showing that the probability of AAP+SOC being cost-effective in this context is close to zero for all values of the cost-effectiveness threshold below the NICE threshold.

The probabilistic sensitivity results of the mean and 95% CI total costs and QALYs are presented here in Table S32. Best practice guidance is that confidence intervals are not reported for ICERs [1] and hence the reporting of the cost-effectiveness acceptability curve.

Table S32. Mean and 95% CI total lifetime costs and QALYs for the two subgroups, by arm, using 25 simulations and 500 iterations.

	**M0 subgroup**		**M1 subgroup**	
	**AAP+SOC**	**SOC-only**	**AAP+SOC**	**SOC-only**
Total lifetime costs (mean) (2017-18 UK £)	95,742	46,957	114,792	45,058
Lower bound for 95% CI	62,974	36,826	75,326	34,345
Upper bound for 95% CI	143,941	59,453	172,256	63,351
Total lifetime QALYs (mean)	7.01	6.51	4.44	2.92
Lower bound for 95% CI	6.09	5.59	3.70	2.42
Upper bound for 95% CI	7.88	7.52	5.30	3.50

References

[1] A. Briggs, M. Sculpher and K. Claxton, Decision Modelling for Health Economic Evaluation, Oxford: Oxford University Press, 2006.
