# Supplementary material for: Cost-utility analysis of adding abiraterone acetate plus prednisone/prednisolone to long-term hormone therapy in newly diagnosed advanced prostate cancer in England: Lifetime decision model based on STAMPEDE trial data
Source: PLoS One. 2022 Jun 2;17(6):e0269192. doi: 10.1371/journal.pone.0269192 (PMC9162346; doi:10.1371/journal.pone.0269192)
Supplement: S10 File — (PDF) [file pone.0269192.s011.pdf]

Cost-utility analysis of adding abiraterone acetate plus prednisone/prednisolone to long-term hormone therapy in newly diagnosed advanced prostate cancer: lifetime decision model in England based on STAMPEDE trial data

Caroline S Clarke*, Rachael M Hunter, Andrea Gabrio, Christopher D Brawley, Fiona C Ingleby, David P Dearnaley, David Matheson, Gerhardt Attard, Hannah L Rush, Rob J Jones, William Cross, Chris Parker, J Martin Russell, Robin Millman, Silke Gillessen, Zafar Malik, Jason F Lester, James Wylie, Noel W Clarke, Mahesh KB Parmar, Matthew R Sydes, Nicholas D James on behalf of the STAMPEDE investigators

* Corresponding author: caroline.clarke@ucl.ac.uk (CSC), Research Department of Primary Care and Population Health, University College London, London, UK.

Supporting Information

File 10

Relating to *Results: Lifetime simulation model: Deterministic abiraterone cost threshold analysis* section of the main manuscript

The BNF cost of abiraterone is likely to be an over-estimate of the price paid by the NHS. The threshold analysis produced results using different reduced daily cost inputs for the price of abiraterone (see Table S33), and trend lines were added to the graph using Excel. This analysis suggested that, with a daily price of £48.84 for 1000mg of abiraterone (50% reduction from base case price), addition of AAP would lie below the NICE upper bound threshold of £30,000/QALY gained for M1 patients. A further reduction to 25% of the BNF price reduced the ICER to below the threshold for all patients, and when the price was reduced to 10% (£9.77/day, comparable to current prices in India), then addition of AAP dominated SOC-only in M0 patients, i.e. it cost less and provided more benefits, and was below the NICE threshold for M1 patients.

The proportion of the BNF price below which AAP+SOC is of equivalent or greater lifetime cost-effectiveness compared to SOC-only was calculated from the linear trendlines shown in Fig S6. These proportions were 29% of the BNF price (£28/day) in the M0 subgroup and 63% of the BNF price (£62/day) in the M1 subgroup.

The linear trendline for M0 costs crosses the x-axis at x=11.7, i.e. if the price of abiraterone were reduced to 11.7% of the current BNF price (£11/day), then the cost per QALY gained would be zero.

Table S33. Results of the threshold analysis, examining the impact of reducing abiraterone daily cost on the mean lifetime incremental cost and therefore the lifetime ICER.

	M0 subgroup	M1 subgroup
Difference in lifetime QALYs	0.33	1.48
**Abi daily cost £97.68, 100% BNF**		
Difference in costs (£)	£48,821	£70,246
ICER (£/QALY)	**£149,748**	**£47,503**
**Abi daily cost £73.30, 75% BNF**		
Difference in costs (£)	£34,999	£52,783
ICER (£/QALY)	**£107,352**	**£35,693**
**Abi daily cost £48.84, 50% BNF**		
Difference in costs (£)	£21,177	£35,319
ICER (£/QALY)	**£64,956**	**£23,884 †**
**Abi daily cost £24.42, 25% BNF**		
Difference in costs (£)	£7,355	£17,856
ICER (£/QALY)	**£22,560 †**	**£12,075 †**
**Abi daily cost £9.77, 10% BNF**		
Difference in costs (£)	-£938 †	£7,377
ICER (£/QALY)	**AAP+SOC dominates †**	**£4,989 †**

†ICER below NICE upper threshold of £30,000/QALY.

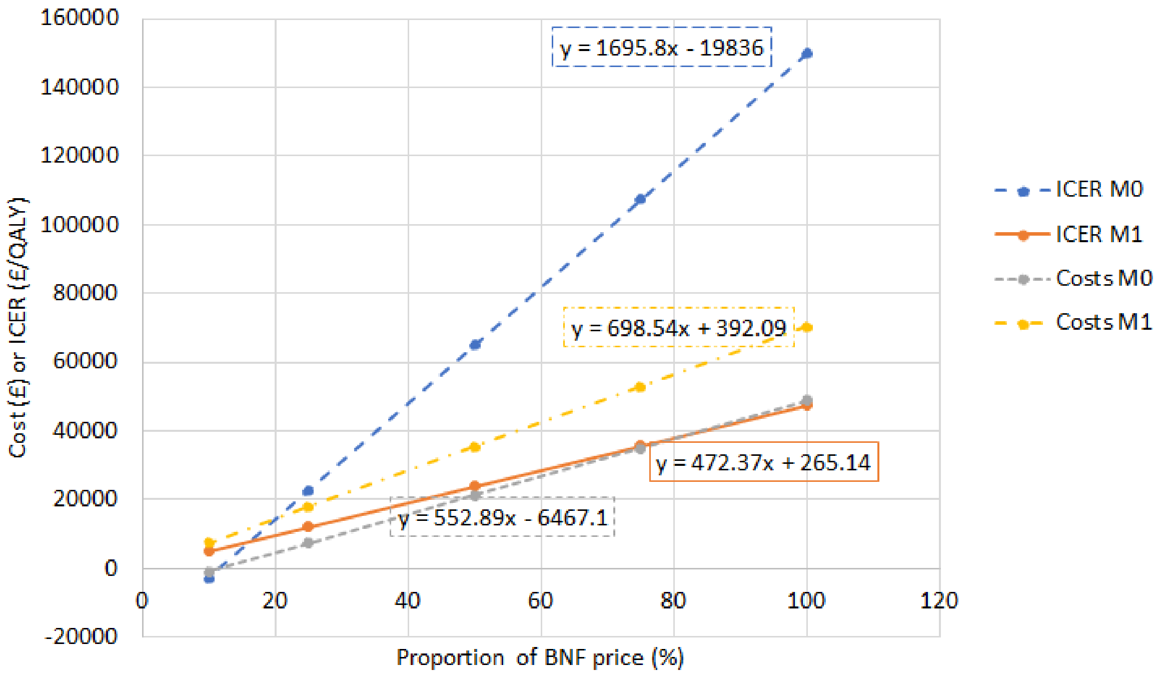

Fig S6. Linear trendlines in the threshold analysis for the ICERs of the two subgroups. The trendline equations are also shown. It can be seen that the lines for the M0 subgroup cross the y-axis (i.e. incremental cost equal to zero) at x=11.7%.
